# Mental Health and Work: A Systematic Review of the Concept

**DOI:** 10.3390/healthcare12232377

**Published:** 2024-11-26

**Authors:** Selma Lancman, Sofia Pinto Bueno de Campos Bicudo, Daniela da Silva Rodrigues, Lilian de Fatima Zanoni Nogueira, Juliana de Oliveira Barros, Barbara Iansã de Lima Barroso

**Affiliations:** 1 Department of Physiotherapy, Speech Therapy and Occupational Therapy, Faculty of Medicine, University of São Paulo, São Paulo 05360-160, Brazil; lancman@usp.br (S.L.); sofia.bueno@fm.usp.br (S.P.B.d.C.B.); 2 Occupational Therapy Course, Federal University of Brasília, Ceilândia Campus, Brasilia 70297-400, Brazil; danirodrigues.to@gmail.com; 3 Department of Occupational Therapy, University of Sorocaba, Sorocaba 18023-000, Brazil; lilian.zanoni@prof.uniso.br; 4 Department of Preventive Medicine, Federal University of São Paulo, São Paulo 01246-903, Brazil; bil.barroso@unifesp.br

**Keywords:** mental health and work, systematic review, concepts and definition of mental health, stress disorders, burnout, public policy

## Abstract

Background: The association between mental health and work has led to multiple meanings and definitions, leading to generalizations and equivalences that do not always reflect similar meanings. Objectives/Methods: To map and analyze the definitions of mental health related to work in the literature and identify the professional practices arising, a systematic review was carried out following PRISMA 2020 guidelines. Six databases were selected for consultation, which was carried out in March 2023. Results: From the search, 26 articles were selected and most of them do not define the concept of mental health, nor the influence of work on mental health–illness processes. Few articles report working conditions and the professional environment as generators of stress. Even if these conditions exist, the studies highlight that individuals already had previous personality traits that made them susceptible to disorders. Conclusions: Failure to adopt clear theoretical–methodological references regarding the concept of mental health and its relationship with work weakens the field and professional practice. Moreover, the literature does not point to changes in work or indicate possibilities for returning to assisted/compatible work, vocational reorientation, or other means of overcoming the problem within work and organizations, with significant impacts on the design of public policies in the field.

## 1. Introduction

Mental health disorders are among the most significant causes of work-related illnesses. In Brazil, for example, they are the third leading cause of absence from work [1]. These disorders end up generating more serious consequences, such as early retirement, burnout, and even increasing work-related suicides in more severe cases.

Mental health is a multi-professional and interdisciplinary field of activity that emerged in opposition to the paradigm of medical psychiatry. It is often associated with the study of and intervention in mental illnesses. Mental health developed first in the 1950s/60s in Europe and North America, which were undergoing important psychiatric reforms. In this new context, mental health practice began to emerge and grow in extra-hospital services, such as outpatient clinics, day centers, private clinics, and companies [2].

The care offered in the field of psychiatry was commonly aimed at people with severe mental disorders. With the advent of new psychotropic drugs and the popularization of psychotherapies and multidisciplinary teams, this care was simultaneously directed at people with mild and moderate psychiatric disorders, especially those related to depression [2].

Mental disorders started to be associated with work in the 1950s in the post-war period when their etiology was debated. This discussion gained momentum from the 1960s/70s, a period of intense industrialization processes and a simultaneous increase in the number of cases of mental illness and the identification of the burden related to absence from work [3,4].

One of the hypotheses of this review is that the notion of mental health used in work-related studies is polysemic. In some of them, it designates “good mental health” as an event opposed to mental illness, the absence of psychic illnesses; in others, the positive idea of happiness and self-fulfillment, processes of resilience and optimism, the capacity for self-realization, and overcoming difficulties are highlighted [5,6].

By acquiring multiple meanings and definitions, the association between mental health and work led to generalizations and equivalences that are not always epistemologically similar. One important aspect refers to the causal relationship between work and the mental health–illness process: sometimes the focus of the issue is centered on people and their weaknesses, while at other times it is on work and its organization. This discrepancy in understanding the problem can lead to conflicting practices—should we develop practices focused on people or centered on work transformations?

These generalizations hinder scientific advances, as they make it difficult to compare and deepen the understanding of the problems, understand their origin, and make possible changes in development that could reduce such problems. From the perspective of the work of professionals in the field, these conceptual uncertainties hinder preventive actions and actions to transform the work environment. Addressing similar concepts that do not interact with each other, focusing the problem solely on individuals, and the absence of concepts that understand that work and its content are fundamental in formulating transformative actions, have prevented us from overcoming the problem.

Although many studies have been conducted and published with mental health and its relationship with work as one of the central topics, related to their title and keywords, in most research, the term does not seem to be explicitly defined and is taken as a holder of unique meaning, as if it were understood in a similar way by everyone. Some explore the topic through medical models, highlighting symptoms and diagnoses of mental disorders according to group VI of ICD-11, such as depression, post-traumatic stress, and anxiety [7,8,9]. It is noteworthy that in ICD-11, within the group [10], burnout was recognized as an occupational disease (CID XI).

In the scientific field and health and work, a substantial gap was observed regarding the lack of clarity in the definition of mental health and its relationships with work, and the lack of a basis for the resulting practices, aspects that motivated this review. Is the definition of mental health based on medical categories? Is well-being synonymous with mental health? Is it possible to define mental health and its relationship with work through the different concepts of stress, which in turn are defined as a set of symptoms? What are the implications of lacking a clear definition of mental health in the field and in health and work practices?

It is understood that the polysemy of the term does not contribute to advances in the area via weakly overlapping concepts with distinct epistemologies and diverse practices without presenting their respective theoretical–methodological bases. Moreover, this does not favor the challenge to studies or the creation of scientific evidence in the approaches and methods used.

Work’s centrality in the construction of adult mental health, the importance of mental health in the workplace, and worker’s health, whether regarding absence, illness, or high levels of distress, reveals a relevant contemporary problem. In the same way that problems related to mental health can lead to sick leave and care, a better understanding of them is also essential for the vocational rehabilitation of people diagnosed with mental problems or on sick leave due to mental disorders.

Thinking about mental health and its relationship with work also means understanding the organizational aspects and management of production processes, and promoting mental well-being at work (which, in this case, is not synonymous with mental health). However, in the literature, most studies focus on people and not on work [11]. In this sense, this review aims to map and analyze the different definitions of work-related mental health in the literature and identify the professional practices arising from the perspectives adopted.

## 2. Materials and Methods

This systematic review was performed under the PRISMA 2020 guidelines [12] and was registered on the International Prospective Register of Systematic Reviews (PROSPERO)—CRD[NN]).

Mapping concepts in systematic reviews is an emerging proposition for producing knowledge that seeks to compile, understand, identify, describe, and characterize the concepts used by a given scientific population, exceeding epistemological questions [13,14].

### 2.1. Eligibility Criteria—Study Selection

The eligibility criteria include: (1) empirical research using qualitative or quantitative approaches, peer-reviewed journal—published journal (not editorials, book chapters, systematic reviews, theoretical papers, etc.); (2) the studied population was exclusively a working population; (3) published in the English language; (4) studies that reported the concept of mental health related to work, worker’s health, and occupational health through the research objective, after reading the titles, abstracts, and full text; (a) to analyze the various concepts of mental health used in health and work research; then (b) to check the similarities in the concept of mental health and oppositions, convergences and divergences in research in this field.

### 2.2. Information Sources

The serial search of articles was performed using the following electronic databases: PubMed Central (PMC), Scopus, Web of Science, Eric, Embase, and PsycINFO. The databases were consulted between 10 and 11 March 2023.

### 2.3. Search Strategy

Two reviewers designed and conducted the search strategy. The terms used in the research were based on the Medical Subject Heading (MeSH) descriptors, and Boolean operators were used between the terms, namely: [(“Mental Health” OR “Health Mental” AND “Occupational Health” OR “Employee Health” OR “Health, Employee” OR “Health Occupational” OR “Worker” OR “Working” OR “Work” AND “Psychopathology”)]. There was a search date limitation from 2012 to 2023.

### 2.4. Selection Process and Data Collection Process

Four researchers independently and blindly scanned the articles for each evaluation step using Rayyan Systematic Review software^®^ 2022. The steps consisted of (1) reading the title and abstract and (2) reading the article in its entirety. Any conflict between the choice and selection of the analyzed material was resolved by a judge. At all stages, a judge was needed to analyze disagreements between researchers.

The articles were then reviewed in depth and coded based on their objective, research design, sample, context, and mental health concept.

### 2.5. Data Items

Rayyan^®^ 2022 software was used for data management, synchronization, import, and storage. The selection process for the research hierarchically included a three-step data collection procedure: (1) analysis and selection by the screening of titles (two researchers), (2) analysis and selection by reading the abstracts (two researchers), and (3) analysis and selection by reading the complete eligible texts (two researchers).

After this step, a structured data extraction form was adopted to organize information on the study’s characteristics, including the following data: title, author and year, country, sample size, and mental health concept. Then, they were reviewed after meeting the inclusion criteria.

### 2.6. Synthesis Methods

For this investigation, the inclusion criteria were that the article should be: (1) from 2012 onwards; (2) written in English; (3) indexed in the selected databases, with full and free full-text availability; (4) from a peer-reviewed journal study; and (5) without restrictions regarding the place of origin of the manuscript. The exclusion criteria were replicated articles that did not address the topic according to the analysis of the title, and abstract and review studies.

At the end of the extraction process, the data were extracted from each publication and summarized to provide a full description of the studies. Finally, the authors reflected on their operationalization to analyze how the literature reports provide an integrated view of the different definitions of mental health, or a lack thereof, in the literature and the type of practices they point to.

### 2.7. Assessing Publication Bias and Evaluation of Certainty

A structured template was developed to extract relevant data from eligible articles. As this is a systematic theory review article, the Critical Evaluation instrument, adapted from the Joanna Briggs Institute, was used to analyze the critical assessment [15].

Regarding selection bias quality, confounding factors, and percentage of results data, the authors decided not to conduct a meta-analysis to present quantitative data. Therefore, we performed a narrative synthesis of the results that shows the heterogeneity of the studies included.

## 3. Results

### 3.1. The Selection of Studies

**Figure 1:**
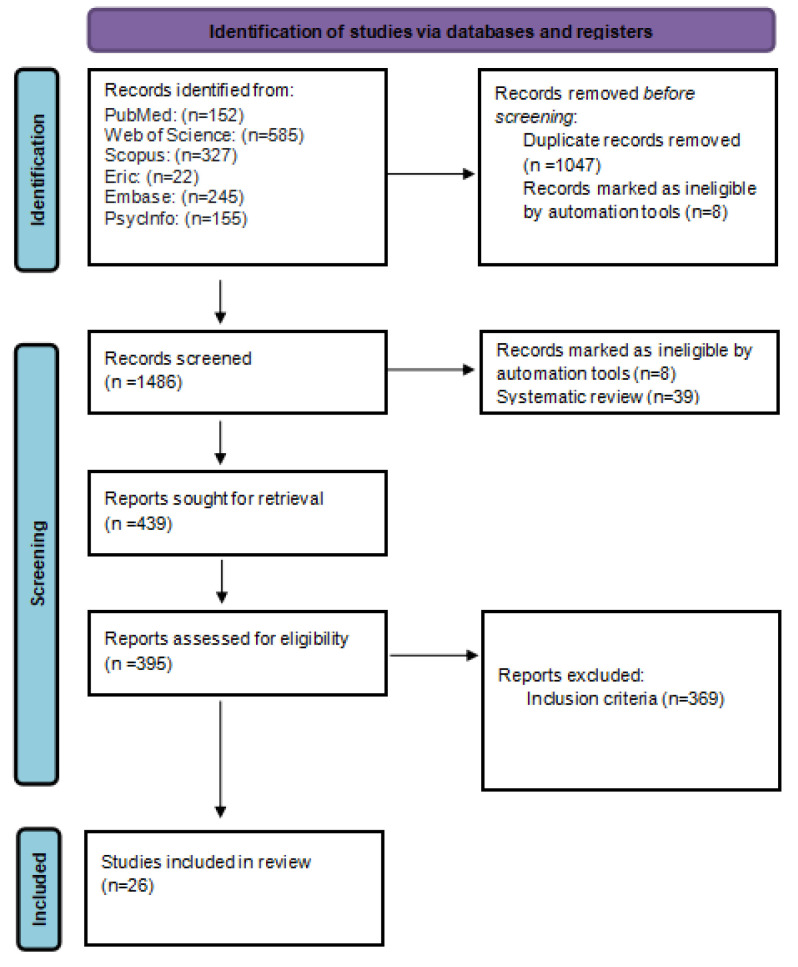
PRISMA 2020 flow diagram for new systematic reviews, which included searches of databases and registers only.

The literature search resulted in 1486 potentially eligible articles, of which 1047 were removed as being duplicates, and 8 were removed using Rayyan^®^. The 439 articles were selected to read the title and summary. After this step, 44 articles were excluded in screening complete articles and after applying the inclusion criteria, leaving 26 studies included in this review. Figure 1 illustrates the PRISMA flow diagram.

### 3.2. Characteristics of the Studies

Regarding the characteristics of the studies, of the total of 14 countries, five studies were conducted in the United States, four in Italy, four in China, three in Spain, two in Canada, two in Brazil, and two in Mexico, and only one study was conducted in other countries, for example (Australia, New Zealand, India, Saudi Arabia, Iran, Ecuador, and the United Kingdom) (See Table 1).

The attributes of the eligible studies (article, description, and critique) are shown in Table 1. Regarding the characteristics of the eligible studies, seven studies associated mental health with the psychopathological profile caused by extreme conditions in the work environment [16,17,18,19,20,21,22]; two studies distinguished between mental illness (psychological symptoms) and positive health (well-being) [23,24]; six compared mental distress with the characteristics of work, the social/economic context, family issues and the demographic context [25,26,27,28,29,30]; two associated mental health with occupational stress [10,31]; and six studies made an association between psychopathologies, such as anxiety, depression and post-traumatic disorder, related to poor working conditions, such as violence and work-related stress [32,33,34,35,36,37]. Some issues, such as the characterization of well-being and resilience in the workplace, were highlighted in three studies [24,27,28].

Although much research occurred during the COVID-19 pandemic, only three articles evidenced clear associations between the stress generated by COVID-19 and mental disorders due to the characteristics of, and working conditions during, the pandemic period [22,23,38].

Twelve of the 26 studies included were conducted and published in 2021: 24, 25, 28, 29, 30, 31, 32, 35, 36, 37, 39, 40; three in 2022: 11, 22, 23; three in 2019: 19, 27, 34; two in 2020: 20, 21; and two in 2017:17, 18. Only one study was published in each of the following years: 2012: 41; 2013: 33; 2015: 26; and 2023: 38.

**Table 1:** Attributes of eligible studies.

Title	Author, Year, and Country	Design and Sample	Mental Health Concept
Anger, personality traits and psychopathological symptoms in subjects exposed to negative interpersonal actions in workplaces: an observational study in a large sample attending a Center for Occupational Stress [10]	Forresi, B. et al., 2022, Italy	A total of 1676 workers attending a Centre for Occupational Stress, 717 males and 959 females. Retrospective study over 3 years. Data collected: clinical, sociodemographic, and occupational.	Mental health is associated with the psychopathological profile (medical classification, disorders) of the sample.
Mental disorders and employment status in the São Paulo Metropolitan Area, Brazil: gender differences and use of health services [16]	França, M.H. et al., 2017, Brazil	General adult population (18+). Population-based cross-sectional study, in which data were collected between May 2005 and April 2007 by trained lay interviewers	Mental health as a synonym for mental disorder DSM-IV (Statistical Manual of Mental Disorders 4th edition). Associated with employment status
Mental health of the prison medical workers (OMWs) and influencing factors in Jiangxi, China [17]	Liu, X. et al., 2017, China	All the prison medical workers in Jiangxi province who were in service were officially hired by the government. Cross-sectional survey with statistical analysis by *t*-test. Survey questionnaires were delivered in each prison and returned once finished	Mental health as a mental disorder, including psychological symptoms and psychological health related to work environment and conditions
Anxiety in the workplace: a comprehensive occupational health evaluation of anxiety disorder in public school teachers [18]	Jones-Rincon, A. and Howar, K. 2018, United States	A total of 3.361 public school teachers, from 46 random school districts in Texas. Online questionnaire—questions about demographics, occupational and psychosocial factors. Hierarchical logistic regression	Mental health is a concept that encompasses different mental disorders, such as GAD and MDD, which can have a negative effect on occupational health.
Psychological status and fatigue of frontline staff two months after the COVID-19 pandemic outbreak in China: A cross-sectional study [19]	Teng, Z. et al., 2020, China	Front-line staff during the COVID-19 pandemic. A total of 2614 participants—community workers (27.5%), health care workers (14.8%), volunteers (21.4%), market administrators (11.2%), and others—including commanders, police and journalists (24.6%). Cross-sectional survey design with anonymous online questionnaire. Sampling was carried out with the snowballing strategy. Tests used for statistical analysis were chi-square and sum-test, along with multivariable analysis with ordinal logistic regression and Superman correlations	Mental health is viewed as psychological problems, including depression, anxiety, and insomnia. It is associated with a poor work environment and conditions and can lead to poor work performance.
Studying the relationship between factors related to stress management of insurance employees of Iran social security organization [20]	Shojaeian, R. et al., 2020, Iran	Three sample groups: one of experts with 15 people, one with 464 employees of the Social and one with Social Security Organization, and one with 232 Managers Survey and questionnaire (3); correlational descriptive research, and data analysis was performed using structural equation modeling with Lisrel software	Mental health associated with mental illness and work fatigue caused by behavioral factors, the personality of workers, and workplace characteristics (stress)
Barriers to mental health-seeking in veterinary professionals working in Australia and New Zealand: a preliminary cross-sectional analysis [21]	Connolly, C.E. et al., 2022, Australia and New Zealand	A total of 408 veterinary professionals, working and over the age of 18. An online survey between June and December 2021, descriptive statistics, and ANOVA with Tukey post hoc comparisons	Mental health is connected to mental ill health and symptoms, associated with psychiatric/psychopathological disorders (depression, anxiety, burnout, and suicidality), related to poor work conditions.
Stress, depression, anxiety, and burnout among healthcare workers during the COVID-19 pandemic: a cross-sectional study in the tertiary center [22]	Jaber, M.J. et al., 2022, Saudi Arabia	A total of 831 participants over 3 months were recruited, 87.6% nurses, 4.2% physicians, and 8.4% allied. health professionals. Cross-sectional, descriptive, and correlational design. It used surveys, questionnaires, and descriptive statistics to analyze the data	Mental health as depression, anxiety, stress, burnout, and other “issues” which. result from a certain profession (in this case healthcare). It also relates said issues with the COVID-19 generated stress
The influence of technology on the mental well-being of STEM teachers at the university level: COVID-19 as a stressor [23]	Navarro-Espinosa, J.A. et al., 2021, Ecuador and Spain	University teachers with a medium of 20 years of working experience (N = 55). Mixed method approach: a cross-sectional study (survey in September–October 2020) and a biometric analysis carried out on 3 databases	Mental health is associated with psychological and psychiatric disorders that influence both psychological and biological health as well as work performance
Anxiety, trauma, and well-being in health-care professionals during COVID-19 first wave in Spain: the moderating role of personal protection equipment availability [24]	Bajo, M. et al., 2021, Spain	A total of 232 healthcare professionals in Spain in the first line of COVID-19 patient care. Measurements using scales and parallel analysis and exploratory factor analysis, ANOVA, and hierarchical regression analysis	Mental health is associated with depression, anxiety, and insomnia. Distinguishes between mental illness (psychological symptoms) and positive health (well-being). Defines mental health as “a set of symptoms of hedonic (emotional well-being) and positivity functioning (psychological well-being and social well-being)”.
Mental health and functioning of female sex workers in Chittagong, Bangladesh [25]	Hengartner, M. P. et al., 2015, India	A total of 259 women sex workers. The comprehensive Composite International Diagnostic Interview was used to assess 12-month prevalence rates of DSM-IV mental disorders, and a comprehensive questionnaire was adapted to explore various factors, such as sociodemographics, working and living conditions, or experiences of abuse.	Mental health associated with the DSM-IV mental disorders related to factors such as sociodemographics, working and living conditions, or experiences of abuse. The one analysis of the current study was mood disorders, anxiety disorders, substance use disorders, and impulse control disorders.
The role of socioeconomic status, occupational health, and job rank on the epidemiology of different psychiatric symptoms in a sample of UK workers [26]	Lopes, B. Kama, C. Jaspal, R. 2019, United Kingdom	A total of 4596 employees collected from the United Kingdom Psychiatric Morbidity among Adults Living in Private Households Survey (age from 16–74 years). Analysis of a data set comprising 8589 people who took part. in extension assessment interviews for the Psychiatric Morbidity among Adults in private Household Survey between March and September 2000	Mental health related to depressive, avoidant, and paranoid symptoms among employees, associated with job rank, household gross income, social class, personal gross income, and socioeconomic group (which were also associated with physical and occupational health
Application of the dual-factor model of mental health among Chinese new generation of migrant workers [27]	Zhang, Que. Lu, J. Quan, P. 2021, China	A total of 515 new-generation migrant workers in the electronics and machinery industries. Questionnaire (survey) followed by descriptive statistics and ANOVA, data collected in October 2015	Classifies mental health into 4 types > mentally healthy, vulnerable, symptomatic but content and troubled (psychopathology may coexist with high levels of well-being, and absence of psychopathology and low well-being may coexist).
Benefits of expressive writing on healthcare workers’ psychological adjustment during the COVID-19 pandemic [28]	ProcacciaR. et al., 2021, Italy	A total of 55 healthcare workers who worked during the COVID-19 pandemic were randomly divided into the intervention or control group. Data were collected from April to June of 2020. Intervention + questionnaires + statistical analysis (descriptive statistics and ANOVA)	Mental health is related to psychological distress, such as depression, insomnia, anxiety, and post-traumatic symptoms, due to work characteristics and social context, but also demographics. Other terms used: mental well-being.
Psychological Impact of the COVID-19 Pandemic on Frontline Health Care Workers During the Pandemic Surge in New York City [29]	Feingold, J.H. et al., 2021, United States	Front-line staff during the COVID-19 pandemic. A total of 6.026 participants—community workers (27.5%), health care workers (14.8%), volunteers (21.4%), market administrators (11.2%) and others. Cross-sectional survey design with anonymous online questionnaire. Sampling was carried out with the snowballing strategy	Mental health is viewed as psychological problems, including depression, anxiety, and insomnia. It is associated with a poor work environment and conditions and can lead to poor work performance
Stress anxiety and depression among gastronomes: association with workplace mobbing and work-family interaction [30]	Machado, I. et al., 2021, Brazil	A total of 160 technologists and bachelors in gastronomy who work or worked in Brazilian commercial restaurants—59.6% women, with an average age of 30.81 years. Observational–analytical, cross-sectional study with a quantitative approach, the data were collected through questionnaires was analyzed using descriptive and inferential statistics.	Mental health is perceived as the presence of stress, anxiety, and depression, associated with labor demands, commands, and mobbing at work, and also affected by family relations.
Unemployment and mental health in community population from a border city in Mexico [31]	Zamorano González, B. et al., 2021, Mexico	Sample of community population, inhabitants of an urban area. The sample consisted of 1351 participants, 43% men and 57% women, with an average age of 41.46 years. Transversal, correlational, and quantitative study, with a home survey	Mental health is connected to psychopathologies (depression, anxiety, or stress) and mental disorders with a relation between work status and mental health (unemployment was related to higher scores in sub-scales of psychopathologies). Mental health is negatively affected during periods of economic distress
Occupational stress and psychopathology in health professionals: an explorative study with the multiple indicators multiple causes (MIMIC) model approach [32]	Iliceto, P. et al., 2013, Italy	A total of 156 health professionals (nurses and physicians) from 3 hospitals in the district of Rome (62 males, 94 females). Survey with questionnaires with a two-tailed *t*-test and chi-square test + structural equation modeling	Mental health connected to stress and multivariate processes linked to personality characteristics, coping processes, and positive and negative work
Psychological strain, depressive symptoms, and suicidal ideation among medical and non-medical staff in urban China [33]	Liu, Y. et al., 2018, China	Randomly selected medical employees of a large hospital (n = 1012) and a second sample including heterogeneous office employees (n = 1052). Self-administered questionnaire to collect data—the same questionnaire was used for both medical and non-medical staff	Mental health as a concept that encompasses psychological strain that can lead to depressive symptoms and suicidal ideation, associated with work organization
Post-trauma psychopathology in journalists subtitle: the influence of institutional betrayal and world assumptions [34]	Dadouch, Z. Lilly, M. 2021, United States	A total of 115 journalists from different organizations in the USA, women and men. Online surveys, clinical phone interviews, and data collection started in February 2017. Chance to draw for 2.50 USD gift cards	Mental health is viewed from the perspective of post-trauma psychopathology associated with work conditions
Psychiatric symptoms and moral injury among US healthcare workers in the COVID-19 era [35]	Amsalem, D. et al., 2021, United States	A total of 267 English-speaking, United States resident health caseworkers, 18–80 years of age. Longitudinal three-wave study that assessed clinical symptoms and moral injury at baseline, 30 and 90 days, between September and December 2020, using questionnaires	Mental health is viewed as mental disorders and symptoms, such as depression, generalized anxiety disorder, post-traumatic stress disorder, and moral injury related to the work environment and conditions such as chronic Ams
Workplace violence and psychopathology in paramedics and firefighters: mediated by posttraumatic cognitions [36]	Setlack, J. et al., 2021, Canada	A total of 117 firefighters, all career full-time, 129 paramedics, making a total of 246 providing complete usable data. Series of online self-report measures through Qualtrics. Those who completed the survey were eligible for financial compensation (500 USD fuel card)	Mental health is understood as psychopathologies and its symptoms such as PTSD, depression, and anxiety related to work conditions and experiences such as violence, traumatic events, and stress
Perceived coworker social support: a protective factor against workplace violence and psychopathologies in paramedic and firefighters [37]	Brais, N. et al., 2023, Canada	A total of 43 firefighters and paramedics. Data were collected from December 2018 to March 2019 using an online self-report survey. Statistical analysis: moderated regressions	Mental health associated. with psychopathologies such as anxiety, depression, and post-traumatic disorder related to poor work conditions (such as violence and stress)
“Healthcare Kamikazes” during the COVID-19 pandemic: purpose in life and moral courage as mediators of psychopathology [38]	Echeverria, I. et al., 2021, Spain and Mexico	A total of 149 healthcare professionals and 56 medical and nursing students. Observational, cross-sectional study of cases and controls. Use of questionnaires	Mental health is associated with mental disorders and high levels of stress, depression, anxiety, and post-traumatic stress symptoms, due to work characteristics and conditions
Anxiety, Depression and Risk of Post-Traumatic Stress Disorder in Health Workers: The Relationship with Burnout during COVID-19 Pandemic in Italy [39]	Ghio, L. et al., 2021, Italy	A total of 731 health care workers from hospitals in Genoa, Italy. Longitudinal project. Structured online questionnaire and validated instruments, from 30 July 2020 to 30 September 2020, and the 2nd assessments after 6 months	Mental health status is determined by psychological and psychiatric disorders, such as anxiety, depression, insomnia, post-traumatic stress disorder, due to extreme working conditions (such as stress)
PTSD and depressive symptoms in 911 telecommunications: the role of peritraumatic distress and world assumptions in predicting risk [40]	Lilly, M.M. and Pierce, H. 2013, United States	A total of171 telecommunicators. The sample was predominantly female and European American, aged 38–85 years, married and/or living with a partner. All participants were currently working in telecommunications with an average of 11.85 years of service. Participants were invited to partake in an online or hard copy survey through different methods. There was no financial incentive to partake in the study	Mental health related to PTSD symptomatology and depressive symptoms due to work-related factors such as exposure to traumatic events and to personality traits such as world assumptions (fundamental assumptions to help organize external input—it can be of 3 types: the world is benevolent, the world is meaningful and the self is worthy). The link between trauma exposure, mental health, and world assumptions

## 4. Discussion

### Definitions of Mental Health and Its Relationship with Work

The relationship between Mental Health and Work is a topic that has gained increasing relevance in recent decades, given its complexity and its impact on the health of working people. Evaluating these concepts is fundamental to advancing the discussion and understanding the dynamics of the world of work. Carrying out a systematic review of the subject makes it possible to gather and critically analyze the available evidence, contributing to the construction of more robust and up-to-date knowledge. Furthermore, by synthesizing information from various studies, the review provides a comprehensive overview of the main theories, practices, and challenges faced by public policies and organizations concerning mental health at work, promoting reflection on the need for effective interventions.

Based on the analysis of the literature studied, it can be seen that studies do not explain the definition adopted when using the term mental health, nor do they highlight its relationships with work accurately. In the 26 selected articles, mental health always appears associated with a field of activity and specialized services [10,16,17,18,19,20,21,22,23,24,25,26,27,28,29,30,31,32,33,34,35,36,37,38,41].

The terms positive health, well-being, positive emotions, and life satisfaction appear as associated with combating stress or enabling individuals to continue working once they are referenced. They are reported freely, without the authors’ concern for clear presentation of definitions, characterizations, epistemological, or theoretical and conceptual bases [7].

In most articles, psychological [18] problems and symptoms of malaise are associated with stress disorders caused by overload [16,17,19,20,21,22,41]. Stress is evident through its symptoms, or some of them: fatigue, mental exhaustion, headaches, eating disorders, irritability, inability to concentrate, anxiety, insomnia, depression, and suicidal ideas. Several articles characterize higher or prolonged forms of stress as causing burnout (professional exhaustion). In these cases, a relationship with work is characterized in the 11th revision of the International Classification of Diseases (ICD 11—WHO) (International Classification of Diseases) [18,22,38].

Work-related overload’s expression appears to be associated with stress disorders. Stress, in turn, is attributed to several aspects, such as family, economic issues (including precarious work), social and demographic issues, or specific events, such as COVID-19 [22], which affected health professionals who were on the front line of treating those affected by the disease. In this explanatory model, the cause of the stress is removed from work and thus attributed to the subject. Some studies analyze constraints at work, such as moral harassment and bullying, warning about the causes of stress and its consequences for mental health, without, however, defining the term exactly and sometimes addressing stress as a synonym for mental health. These generalizations result in removing the force and consequences of harassment and bullying [42,43].

Stress is sometimes also associated with ethical causes, when the worker is forced to act in disagreement with their principles. Still, these studies point to a definition of stress from a psychiatric medical perspective, based on quantitative data and medical categories. It is also noteworthy that behavioral and cognitive theories inspire stress theories; aspects such as satisfaction, reward, and capacity for work only appear in recent literature in this field [10,31].

For some authors, stress is characterized as a physiological response associated with factors internal or external to individuals. In the literature, it is presented as a cause or accelerator of already existing mental illnesses. Post-traumatic disorders, associated with stress and reported in ICD 10 and 11, are caused by a specific critical event, which can produce prolonged consequences and reactions. However, how the relationship between work and stress occurs is not consensual [7,10,16,18,21,24,27,37]. Although scholars agree that stress reduces occupational performance, it is more often associated with individuals and not with work activity.

Few articles report working conditions and the professional environment as stress generators. Even if these conditions exist, the studies highlight that individuals already had personal traits and a lack of resilience, which favored the condition or its deterioration. There was even some displacement of the problem onto the worker due to a supposed weakness that would favor the development of symptoms. In this sense, workers with depressive or anxious traits are highlighted as those with lower performance and more absence from work [18,22,25,28,37,39]. Work characteristics can improve or deteriorate performance, but ultimately this is primarily related to the characteristics of the individual.

However, while there are criticisms of the stress model that sometimes disregards work and its impacts on health, some articles also point to work and its implications, for example, by linking the psychopathology of work with ergonomics and other disciplines that correlate with stress [10,20,22,23,28,30,32,36,39] Among these, we highlight the lack of focus on subjective aspects related to the organization of work and the importance of work in the psychic construction of individuals. These studies are mainly linked to the psychodynamics of work [39].

Some authors seek to relate mental disorders to psychosocial risks, in which case they use epidemiological instruments and data to measure these through questionnaires and checklists. Others use a positive mental health perspective: words such as well-being and resilience appear to be associated with mental health [10,44]. These are primarily reductionist approaches and are incapable of considering the multiplicity and complexity of the relationship between work activity and mental health production [44,45].

## 5. Conclusions

This systematic review sought to map and analyze the different definitions of work-related mental health in the literature, and to identify the professional practices resulting from the perspectives adopted.

Although the literature in the area uses the term mental health in titles and descriptions (which is why it appeared in the search), and sometimes in the body of the text, this term/conceptualization is not the focus of the work and is not even discussed. What comes up for debate are mental illnesses or psychiatric and psychological symptoms, mostly related to stress. The concept of mental health and its relationship with work is trivialized, as if there were a transparent, homogeneous, and universal definition, which does not need to be explained and opens space for readers to define it in their own way, weakening the theoretical and practical basis of the field and reproducing weaknesses and gaps. In general, when defining the concept of mental health, articles do not consider different approaches or end up defining stress as synonymous with mental health, reducing the concept to one of its symptoms.

The approaches in practically all articles suggest that the leading cause of stress is the workers’ previous characteristics, and these workers need medical or psychological care, generally based on cognitive therapies. This way of thinking does not consider that changes in the conditions and organization of work should be introduced to reduce stress factors, including workload, a problem which ends up feeding back into overwork, stress, absence from work, and new illnesses in potential.

In light of the increasing number of work-related mental health issues and their consequences for workers’ health, this article aims to identify the weaknesses and ambiguities in the definitions of the problem, as well as in the practices associated with it, as discussed in the literature and applied across different countries. Our goal is to raise awareness within the academic community about the importance of standardizing concepts in order to facilitate more accurate comparisons between countries, as well as to highlight the need for organizational changes that could help prevent mental health issues in the workplace.

By addressing the issues raised in various studies on the topic and based on the evidence presented in this article, we believe we are making an original contribution to the debate. We aim to draw attention to the necessity of not separating workers from the context in which they perform their duties, emphasizing that mental health in the workplace should be treated as an integral part of management and the organization of labor practices.

Given the lack of emphasis on the intersection and dialectical relationship between work and mental health in the literature reviewed, other directions in research and practice would be advanced through the development of a broader debate in the international scientific community. Such a debate could favor the creation of consensus regarding the understanding of the concept and the relationship between work and mental health. It is suggested that labor, health, and social security legislation, in other words occupational health and safety policies, take into account the role of work in mental health and illness processes, which would encourage the adoption of practices that could take into account the work situation and its components, such as organizational and relational aspects and the content of the work itself, to the detriment solely and exclusively of approaches aimed at sick workers. In this way, we could effectively contribute to the development of prevention practices and, above all, the promotion of mental health through work. The global guidelines published by the World Health Organization, in partnership with the International Labor Organization, provide us with important clues for building good practices in this field [46].

The results of this review study have some strengths and some limitations, including issues involving the underuse of the terms mental health and stress, which have become complex to analyze. While many studies presented levels of stress considered as the prevalence of depression, burnout, and stress, other studies used the same names with different meanings, thus establishing different limits.

The literature does not point to changes in work and does not even indicate possibilities for returning to assisted or compatible work, vocational reorientation, or other means of overcoming the problem within the scope of work and organizations, which has significant impacts on the design of public policies in the field.

## Data Availability

Not applicable.
